# Serglycin: At the Crossroad of Inflammation and Malignancy

**DOI:** 10.3389/fonc.2013.00327

**Published:** 2014-01-13

**Authors:** Angeliki Korpetinou, Spyros S. Skandalis, Vassiliki T. Labropoulou, Gianna Smirlaki, Argyrios Noulas, Nikos K. Karamanos, Achilleas D. Theocharis

**Affiliations:** ^1^Laboratory of Biochemistry, Department of Chemistry, University of Patras, Patras, Greece; ^2^Division of Hematology, University Hospital of Patras, Patras, Greece; ^3^Technological Educational Institute, Larissa, Greece

**Keywords:** proteoglycans, serglycin, chondroitin sulfate, inflammation, malignancy

## Abstract

Serglycin has been initially characterized as an intracellular proteoglycan expressed by hematopoietic cells. All inflammatory cells highly synthesize serglycin and store it in granules, where it interacts with numerous inflammatory mediators, such as proteases, chemokines, cytokines, and growth factors. Serglycin is implicated in their storage into the granules and their protection since they are secreted as complexes and delivered to their targets after secretion. During the last decade, numerous studies have demonstrated that serglycin is also synthesized by various non-hematopoietic cell types. It has been shown that serglycin is highly expressed by tumor cells and promotes their aggressive phenotype and confers resistance against drugs and complement system attack. Apart from its direct beneficial role to tumor cells, serglycin may promote the inflammatory process in the tumor cell microenvironment thus enhancing tumor development. In the present review, we discuss the role of serglycin in inflammation and tumor progression.

## Introduction

Proteoglycans (PGs) are complex macromolecules consisted of a core protein covalently linked with glycosaminoglycan (GAG) chains named chondroitin sulfate (CS), dermatan sulfate (DS), keratan sulfate (KS), heparin (HP), and heparan sulfate (HS). GAGs are negatively charged polysaccharides comprised of repeating disaccharides of acetylated hexosamines (*N*-acetyl-galactosamine or *N*-acetyl-glucosamine) and mainly by uronic acids (d-glucuronic acid or l-iduronic acid) being sulfated at various positions. KS is composed of disaccharides containing *N*-acetyl-glucosamine and galactose (1). PGs are synthesized by all cells and distributed in all tissues participating in physiological functions and pathologic conditions. According to their localization they can be divided in three main groups, the cell-surface associated PGs, such as syndecans and glypicans, the matrix secreted PGs (e.g., versican, decorin, perlecan) and the intracellular PGs, with serglycin being the only characterized member of this subfamily to date (1). Numerous studies have demonstrated significant modifications in PG expression in the tumor microenvironment and their contribution to carcinogenesis (1, 2). The type and fine structure of GAG chains attached to PGs are markedly affected in the context of malignant transformation as a result of the altered expression of GAG-synthesizing enzymes (3). Structural modifications of GAGs may facilitate tumorigenesis in various ways, modulating the functions of PGs (3).

**Figure 1:**
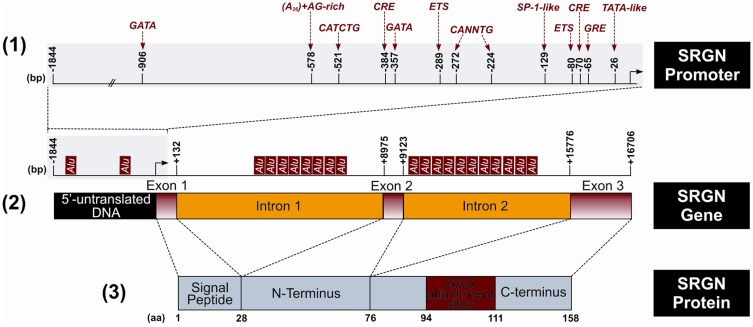
**Schematic representation of the structure of human serglycin (SRGN) gene and protein**. (1) Putative regulatory sites in SRGN promoter. Glucocorticoid response element (GRE), specificity protein-1 (SP-1). (2) Location of the *Alu* elements in the SRGN gene. (3) Encoded SRGN core protein. (bp, base pairs; aa, aminoacid).

Serglycin has been initially considered as a hematopoietic PG present in intracellular secretory compartments. Recent studies demonstrated that serglycin is expressed by a variety of cell types and mediates crucial functions in both normal and pathological conditions (4, 5). Rat L2 yolk sac tumor serglycin was the first PG gene to be cloned and remains until today the smallest known core protein (18 kDa) (6). In human, serglycin consists of a small core protein (158 amino acids) containing eight serine/glycine repeats (Figure 1). Each serine of this repeat region is a potential GAG attachment site. The size of the PG varies according to the GAG chain length, number, and type (7). Serglycin is expressed in all normal hematopoietic cells and hematopoietic tumor cell lines (7–17). In mast cells, eosinophils, neutrophils, and platelets, serglycin is stored together with other bioactive molecules in granules and is secreted upon activation. Serglycin is constitutively secreted by lymphocytes and many hematopoietic tumor cell lines (7, 8, 16). Serglycin is also expressed in non-hematopoietic cells including pancreatic acinar cells (18), chondrocytes (19), smooth muscle cells (20, 21), endothelial cells (20, 22, 23), fibroblasts (20, 24, 25), F9, and NCCIT teratocarcinoma cells (26, 27), murine embryonic stem cells (28), murine uterine dedicua cells, parietal endoderm, and fetal liver but not yolk sac hematopoietic cells (29). Recently, it was demonstrated that serglycin is highly expressed by aggressive nasopharyngeal cancer cells (30). The type and sulfation of GAG chains attached to the serglycin core protein, varies between different species and cell types (4, 8, 16, 17, 22, 23, 31–43). For example, serglycin expressed by human mast calls has been shown to contain both HP and CS chains enriched in disulfated disaccharides [being sulfated at C4 and C6 of *N*-acetyl-galactosamine (CS-E)] attached in separate core proteins, whereas mouse mast cells synthesize a hybrid HP/CS-E serglycin. In leukocytes, platelets, myeloma, and endothelial cells, serglycin contains CS chains that are mainly sulfated at C4 of *N*-acetyl-galactosamine (CS-4). Interestingly, the sulfation pattern of CS present in serglycin is regulated during differentiation of monocytes to macrophages (44, 45). In macrophages serglycin is substituted in a higher degree with CS-E compared to monocytes which contain CS-4 chains. The amount of CS-E expressed by macrophages can be further increased upon activation of differentiated macrophages with phorbol 12-myristate 13-acetate (PMA) (46).

## Structure and Regulatory Elements of the Human Serglycin Gene

The human serglycin gene is located in chromosome 10q.22.1 (47, 48) and is consisted by an approximately 1.8 kb of 5′-flanking DNA, three exons, which are separated by two introns of 8.8 kb (intron 1) and 6.7 kb (intron 2) (49, 50). The 5′-untranslated mRNA and the hydrophobic 27 aminoacid signal peptide of the translated protein are encoded in the first exon, whereas the second exon encodes a 49 aminoacid peptide that represents the amino-terminus of the mature serglycin core protein. Finally, the larger exon 3 codes a 82 aminoacid peptide that contains the GAG attachment region, the carboxy-terminus, and the 3′-untranslated mRNA region (49) (Figure 1). An alternative spliced variant of serglycin lacking exon 2 has been detected in neutrophils and in low levels in HL-60 and may be related with the maturation of promyelocytes to form segmented neutrophils (15). Several putative regulatory sites are present in the 5′-flanking region with E-26 specific family of transcription factors (ETS) site (−80) and the cyclic AMP response element (CRE) half site at −70 to be the most important regulatory elements for constitutive expression (51) (Figure 1). The CRE site is also important for the induced expression of serglycin after treatment with PMA and dibutyryl cyclic AMP (dbcAMP) (51). ETS regulatory elements interact with ETS1 and Friend leukemia integration 1 transcription factor proteins (FLI1). The expression of serglycin was shown to be up-regulated in a number of leukemic cell lines, ones that coincidentally have been shown to express high levels of ETS1 and FLI1 (9, 52). The ETS genes encode transcription factors that play important roles in hematopoiesis, angiogenesis, and organogenesis. In the intron 1 a commonly conserved 70 bp Donehower element is found that may has a *cis*-acting functional role (49). The serglycin gene has also 21 *Alu* elements, with two of them being located in the 5′-flanking region, 8 in the intron 1, and 11 in the intron 2 (49) (Figure 1). *Alu* elements represent one of the most successful of all mobile elements and are primate specific. *Alu* element inserts in or near a gene have the potential to influence expression of that gene in several ways (53). The expression of serglycin in different cell lines depends also on the methylation status and the presence of DNaseI hypersensitivity sites (DHSS) within the serglycin gene (49, 54). Cell-specific DHSS sites have been found in the promoter region, exon 2 and introns of serglycin gene in hematopoietic and endothelial cells (51, 54). These sites are short chromatin regions with disturbed nucleosome formation that have increased sensitivity to factors interacting with DNA and regulate transcription. Several of the DHSS appear to be located well within or very close to *Alu* repeats and this association may play a role in the expression of serglycin (54).

## Binding Partners of Serglycin

**Figure 2:**
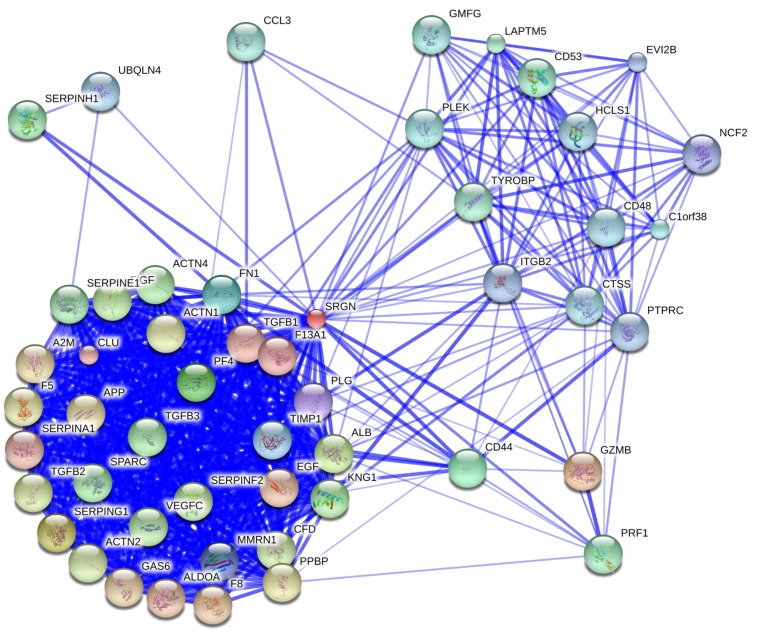
**Overlay of predicted functional partners for human serglycin (SRGN)**. Molecules have been classified according to their confidence score (higher to lower, confidence score >0.48): GZMB, serpin peptidase inhibitor (SERPING1), albumin (ALB), platelet factor 4/CXCL4 (PF4), CD44, fibronectin 1 (FN1), tissue matrix metalloproteinase inhibitor 1 (TIMP1), multimerin 1 (MMRN1), plasminogen (PLG), clusterin (CLU), coagulation factor XIII, A1 polypeptide (F13A1), TGFB1, serpin peptidase inhibitor, clade A member 1 (SERPINA1), epidermal growth factor (EGF), aldolase A (ALDOA), coagulation factor VIII (F8), growth arrest-specific 6 (GAS6), alpha-2-macroglobulin (A2M), amyloid beta (A4), precursor protein (APP), CXCL7 (PPBP), coagulation factor V (F5), actinin, alpha 1 (ACTN1), TGFB2, actinin, alpha 2 (ACTN2), complement factor D (CFD), serpin peptidase inhibitor, clade F (SERPINF2), c-fos induced growth factor/vascular endothelial growth factor D (FIGF), vascular endothelial growth factor C (VEGFC), kininogen 1 (KNG1), actinin, alpha 4 (ACTN4), TGFB3, secreted protein, acidic, cysteine-rich/osteonectin (SPARC), serpin peptidase inhibitor, clade E (SERPINE1), serpin peptidase inhibitor, clade H (SERPINH1), PRF1, LAPTM5, TYRO protein tyrosine kinase binding protein (TYROBP), CD53, CCL3, glia maturation factor, gamma (GMFG), pleckstrin (PLEK), THEMIS2/thymocyte-expressed molecule involved in selection protein 2 (C1orf38), cathepsin S (CTSS), CD48, HCLS1; UBQLN4; protein tyrosine phosphatase, receptor type, C (PTPRC), integrin, beta-2 (ITGB2), ecotropic viral integration site 2B (EVI2B), neutrophil cytosolic factor 2 (NCF2) (http://string-db.org/).

Several studies have demonstrated that serglycin is capable to interact with biological important molecules (summarized in Table 1). The binding is mediated either through GAG chains or core protein or both moieties are required for high affinity binding to serglycin. CS chains of serglycin mediate the binding to CD44 (55), whereas CS-4 chains with a high proportion of 4-sulfated disaccharides (more than 87%) are required for binding to complement components C1q and mannose binding lectin (MBL) (56). Although CS-4 chains are crucial for binding of serglycin to MBL (56) and collagen type I (57), the overall structure may also be important for high affinity binding. This is in agreement with previous studies where the CS-4 chains were essential for binding of serglycin to molecules, such as fibronectin and collagen, chemokine (CXC motif) ligand 4 (CXCL4), chemokine (C-C motif) ligand 3 (CCL3), bone morphogenetic protein (BMP)-like protein, lysozyme, granzyme B (GZMB), perforin (PRF1), and hydroxyapatite although the intact serglycin molecule might interact more efficiently with these molecules (12, 16, 58–63) (Table 1). HP or/and CS-E chains of serglycin in mast cells mediate its binding to chymases (64, 65) and tryptases (65–67) (Table 1). Serglycin colocalizes with carboxypeptidase A (CPA) (68), serotonin, histamine (69), and dopamine (70) in mast cell granules, CPA in pancreatic acinar cells (18), neutrophil elastase in neutrophils (71) and U937 promonocytes (72), tissue-type plasminogen activator (tPA) (23) and chemokine growth-related oncogene-alpha (GRO-α/CXCL1) in endothelial cells (22). Serglycin is capable to interact with matrix metalloproteinases (MMPs), such as MMP13 (19) and proMMP9 through its core protein (73, 74) (Table 1). By using stringent high-throughput yeast two-hybrid system interactions, novel serglycin ligands, such as centrosomal protein 70 kDa (CEP70), BCL2-associated athanogene 6 (BAG6), proline/serine-rich coiled-coil 1 (PSRC1), ubiquitin-protein ligase E3 component n-recognin 4 (UBR4), ubiquilin 4 (UBQLN4), and small glutamine-rich tetratricopeptide repeat (TPR)-containing alpha (SGTA) have been identified (75–77) (Table 1). Among them CEP70 and PSRC1 participate in microtubules formation and assembly of mitotic spindle (78, 79), whereas SGTA and BAG6 play key roles in quality control processes for newly synthesized proteins via their ubiquitination and proteasome degradation (80). Recently, it was shown that BAG6 is not only involved in proteasome core particle assembly but also has a key role in efficient regulatory particle assembly by directly associating with precursor regulatory particles (81). UBR4 and UBQLN4 are also involved in the proteasomal degradation of proteins. UBR4 is an E3 ubiquitin-protein ligase that mediates polyubiquitination of low-abundance regulators and selective proteolysis through the proteasome but is also associated with cellular cargoes destined to autophagic vacuoles and degradation through the lysosome (82, 83). UBQLN4 binds either to ubiquitinated or not proteins and promotes their proteasomal degradation (84) but also mediates the recruitment of ubiquilin1 to autophagosomes (85). In another study, Wu et al. (86) constructed a protein functional interaction network that suggests association of serglycin with known and novel proteins, such as Rho GDP dissociation inhibitor (GDI) beta (ARHGDIB), CCL3, lysosomal protein transmembrane 5 (LAPTM5), arachidonate 5-lipoxygenase-activating protein (ALOX5AP), chemokine (C-X-C motif) receptor 4 (CXCR4), beta-2 microglobulin (B2M), moesin (MSN), hematopoietic cell-specific Lyn substrate 1 (HCLS1), GZMB, RAP1B, member of RAS oncogene family, major histocompatibility complex, class II, DR alpha (HLA-DRA) (see Table 1). A summary of serglycin interactions is available in http://www.ebi.ac.uk/Tools/webservices/psicquic/view/main.xhtml?query=srgn. A large number of candidate molecules might associate with serglycin according to STRING database. A diagram with predicted functional partners for serglycin (confidence score >0.48) is given in Figure 2 (http://string-db.org/).

**Table 1:** **Overview of serglycin binding partners**.

Molecule	Interaction type (Reference)	Major functions
**CELL-SURFACE RECEPTORS, CHEMOKINES, CYTOKINES, AND COMPLEMENT COMPONENTS**		
CD44	Physical association (55)	Cell signaling, adhesion, migration and, lymphocyte activation, hematopoiesis, tumor metastasis
CD53	Computational (experimental knowledge based) (86)	Cell signaling, development, activation, growth, and motility. Activation of leukocytes
HLA-DRA	Computational (experimental knowledge based) (86)	Antigen presentation, immune system response
CXCR4	Computational (experimental knowledge based) (86)	Chemotaxis and activation of leukocytes, hematopoietic stem cell homing, cancer cell metastasis
CXCL1	Colocalization (22)	Chemotaxis, inflammation, angiogenesis, tumor growth
CXCL4	Physical association (60)	Platelet aggregation, chemotaxis, hematopoiesis, angiogenesis, immune system response
CCL3	Physical association (60)	Chemotaxis, inflammation
BMP-like protein	Physical association (60)	Bone and cartilage formation
C1q	Physical association (56)	Complement activation
MBL	Physical association (56)	Complement activation
**MATRIX MOLECULES/COMPOUNDS**		
Collagen I	Physical association (57)	Matrix organization, cell adhesion
Fibronectin	Physical association (12, 58, 59)	Matrix organization, cell adhesion, and migration
B2M	Computational (experimental knowledge based) (86)	Associates with MHC class I molecules. Implicated in amyloidosis and multiple myeloma
Hydroxyapatite	Physical association (16)	Bone formation
**PROTEOLYTIC ENZYME, PORE FORMING PROTEINS**		
Lysozyme	Physical association (60)	Anti-microbial activity
GZMB	Physical association (61–63)	Proteolysis, cell death
PRF1	Physical association (61–63)	Pore formation, cell death
Chymases	Physical association (64, 65)	Proteolysis as part of host defense and inflammation
Tryptases	Physical association (65–67)	Proteolysis as part of host defense and inflammation
CPA	Colocalization (68)	Proteolysis as part of host defense and inflammation
Elastase	Colocalization (71)	Proteolysis as part of host defense and inflammation
tPA	Colocalization (23)	Fibrinolysis. Proteolysis in physiological conditions and diseases
MMP13	Physical association (19)	Proteolysis in physiological conditions and diseases
proMMP9	Physical association (73, 74)	Proteolysis in physiological conditions and diseases
**NEUROTRANSMITTERS**		
Serotonin	Colocalization (69)	Neurotransmission, vasoconstriction, thrombosis
Histamine	Colocalization (69)	Neurotransmission, endothelium permeabilization, inflammation
Dopamine	Colocalization (70)	Neurotransmission
**INTRACELLULAR PROTEINS**		
CEP70	Physical association (75–77)	Microtubules organization
BAG6	Physical association (75–77)	Quality control of proteins, proteasome assembly and degradation, T cell response
PSRC1	Physical association (75–77)	Mitotic spindle assembly
UBR4	Physical association (75–77)	Ubiquitination, proteasomal, and lysosomal degradation
UBQLN4	Physical association (75–77)	Binds ubiquitinated proteins, proteasomal degradation
SGTA	Physical association (75–77)	Cochaperone, quality control of proteins
ARHGDIB	Computational (experimental knowledge based) (86)	Cell signaling, proliferation, cytoskeletal organization, and secretion. Cancer invasion and metastasis
LAPTM5	Computational (experimental knowledge based) (86)	Lysosomal destabilization, cell death
ALOX5AP	Computational (experimental knowledge based) (86)	Leukotriene synthesis, inflammation
MSN	Computational (experimental knowledge based) (86)	Links plasma membranes with actin cytoskeleton. Cell signaling and movement
HCLS1	Computational (experimental knowledge based) (86)	Cytoskeleton remodeling, leukocyte chemotaxis, and activation
RAP1B	Computational (experimental knowledge based) (86)	Cell signaling, adhesion, growth, and differentiation

## Physiological Roles of Serglycin in Inflammation

Serglycin synthesized by inflammatory and stromal cells is secreted either constitutively or in a regulated manner. Serglycin secretion can be induced in several cell types upon external inflammatory stimulation. The biosynthesis of serglycin is up-regulated by liposaccharide (LPS) in macrophages (87), tumor necrosis factor (TNF) in endothelial cells (23) and adipocytes (88) and interleukin 1β (IL-1β) in smooth muscle cells (21).

**Figure 3:**
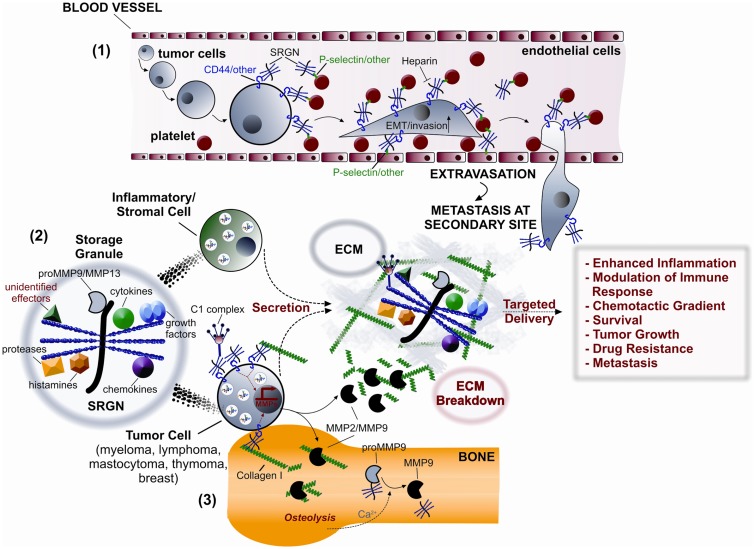
**Potential roles of serglycin (SRGN) in inflammation and malignancy**. (1) Tumor cells and platelets secrete SRGN, which is subsequently bound on their surface via CD44 and/or other receptors, such as P-selectin. Direct and/or indirect (via SRGN) platelet-tumor cell contacts induce EMT, enhance invasiveness, and promote metastasis by binding to P-selectin and/or other receptors present on endothelial cells. (2) SRGN synthesized by inflammatory/stromal and tumor cells is either constitutively secreted or transported to storage granules for subsequent regulated secretion. SRGN-bound molecular effectors (cytokines, chemokines, complement components, growth factors, proteases, MMPs, histamines, and others) are then specifically delivered to target cells, thus controlling inflammation and tumor progression. (3) SRGN present on tumor cell surface promotes their adhesion to collagen I inducing the expression of MMP2/MMP9 resulting in bone destruction and peritumorous ECM breakdown.

The generation of serglycin^−/−^ mice has demonstrated a wide impact of serglycin on the functional properties of immune cells. In inflammatory cells serglycin is localized in secretory granules and vesicles participating in crucial roles in intracellular storage and secretion of bioactive molecules (Figure 3). In mast cells, serglycin is implicated in the storage of granule-localized proteases such as chymases, tryptases and CPA (34, 68), histamine, serotonin, and dopamine in mast cells (69, 70). Serglycin forms complexes with mast cell proteases modulating their activities. Several studies have addressed functions linked to HP, the major GAG component of serglycin, in mast cells. HP/chymase complexes bind to HP-binding substrates of the enzyme thus presenting them to chymase and enhancing their proteolysis (89). HP on serglycin was shown to significantly block the inhibition of chymase by natural inhibitors such as alpha 1-protease inhibitor, alpha 1-antichymotrypsin, alpha 2-macroglobulin, and soybean trypsin inhibitor (90, 91). HP is also involved in the formation of active tryptase tetramers (92, 93). It is suggested that serglycin apart from its protective role for partner molecules is also linked to transport to target sites, where proteases are released to perform their functions (94).

Serglycin mediates the storage of GZMB but not of granzyme A in cytotoxic T lymphocytes (CTLs) (95). It is also essential for the storage of elastase in azurophil granules of neutrophils. In contrast, serglycin is not involved in the storage of other granule components such as cathepsin G and proteinase 3 in these cells (71). Similarly, knockdown of serglycin in platelets results in defective storage of CXCL4, CXCL7, and platelet-derived growth factor (PDGF) (96). The impaired storage of mast cell proteases, neutrophil elastase, GZMB in CTLs and CXCL4 in platelets was not due to altered mRNA levels coding for these molecules, suggesting a crucial role for serglycin on storage rather than mRNA expression. The ability of serglycin to promote storage of granule components is suggested to be due to specific electrostatic interactions between the sulfated GAG chains and the basically charged regions of secretory granule components (95). The stable expression of granule components followed by their diminished protein levels in serglycin knockdown cells and the association of serglycin with SGTA, BAG6, UBR4, and UBQLN4, which are involved in quality control during biosynthesis and degradation of proteins, might suggest a regulatory role for serglycin in cytoplasmic quality control machinery.

Serglycin is also involved in apoptosis and immune regulation. Diminished storage of mast cell proteases in serglycin^−/−^ mice resulted in reduced sensitivity of mast cells to apoptosis as a consequence of the reduced granule damage, reduced release of proteases into the cytosol, and defective caspase-3 activation (97). Mast cells lacking serglycin expression preferentially died by necrosis rather than apoptosis and the necrotic phenotype of serglycin^−/−^ mast cells was linked to defective degradation of poly(ADP-ribose) polymerase-1 (98). Serglycin binds to GZMB and acts as a vehicle for its delivery from CTLs into target cells (62, 63). The impaired storage of GZMB in CTLs and NK cells in serglycin^−/−^ mice may affect their potential to promote killing of neighbor cells (95, 99). Interestingly, serglycin^−/−^ mice infected with lymphocytic choriomeningitis virus are capable to clear virus as efficient as wild type animal but the contraction of the CD8^+^ cells is delayed in serglycin^−/−^ mice, and this could be attributed to sustained proliferation of the CD8^+^ cells. The impaired storage of elastase in neutrophils is possibly associated with the reduced capacity of serglycin^−/−^ mice to clear *Klebsiella pneumoniae* infection (71), whereas after *Toxoplasma gondii* infection only delayed recruitment of neutrophils and lower levels of IL-6 secretion were demonstrated at early stages of the infection without significant immune response impairment (100).

Another interesting implication of serglycin in the regulation of immune system is its ability to inhibit complement system activity. Serglycin isolated from multiple myeloma (MM) cells inhibits the classical and the lectin pathways of complement system through binding to C1q and MBL. CS-4 chains with a high proportion of 4-sulfated disaccharides are required for the interactions with complement proteins (56). CS-E and in a lower extent HP compete with CS-4 chains of serglycin for binding to C1q, whereas only CS-E competes for binding to MBL. Serglycin secreted by various immune cells that carries CS-E or/and HP is more likely capable to regulate the activity of both pathways (56).

In a recent study serglycin was found to be among the most abundantly expressed genes in epicardial adipose tissue and was up-regulated with pro-inflammatory genes such as IL-1β, IL-6, IL-8, and chemokine receptor 2 (CCR2) (88). In human umbilical vein endothelial cells (HUVECs) the storage of CXCL1 in secretory vesicles to the apical side was partly depended on serglycin, whereas upon stimulation with IL-1β an increased colocalization of the two molecules inside the vesicles before secretion was observed suggesting a possible involvement of serglycin in inflammatory conditions (22). The presence of serglycin in platelets may be also important for these cells to fully display their pro-inflammatory properties. In platelets, serglycin associates with fibronectin, PDGF, CXCL7, CXCL4, RANTES/CCL5, and CCL3 and its absence leads to reduced storage of PDGF, CXCL4, and CXCL7 in α-granules, contributing to defective platelet aggregation and leukocyte activation (96). Serglycin expression is elevated in the dermal vessels in dermatomyositis, a chronic inflammatory disease of the skin (101). In addition, mRNA expression of serglycin is induced upon UVB radiation and IL-1α exposure in cultured human dermal fibroblasts suggesting that serglycin may participate in dermal inflammation (25).

## Expression and Biological Roles of Serglycin in Malignancies

Although the main function of serglycin in the biology of immune cells is the proper formation of secretory granules and vesicles as well as the storage and secretion of several components in the extracellular matrix (ECM), the role of serglycin in malignancies is rather intriguing. Serglycin seems to participate in tumor development in a manner that at least partially requires interactions between tumor cells and their microenvironment (102) (Figure 3).

Serglycin with different GAG chains and sulfation patterns is expressed in numerous human hematopoietic and non-hematopoietic tumors (8–17, 26, 27, 30, 55, 103). Interestingly, the megakaryocytic tumor cells synthesize a hybrid CS/HS serglycin (12). The expression levels of serglycin may vary during hematopoietic cell differentiation (9, 14, 15) and is constitutively secreted in the ECM in hematopoietic and solid tumors (16, 30). Serglycin has been proposed as a selective biomarker for acute myeloid leukemia compared to Philadelphia chromosome-negative chronic myeloproliferative disorders since it is highly expressed only by leukemic blasts of patients with acute myeloid leukemia and not in acute lymphocytic leukemia (10). Expression of serglycin has been demonstrated in a variety of lymphoma, myeloma, mastocytoma, and thymoma cells. In these cells the presence of CS-4 or CS-6 side chains of serglycin are required for binding to CD44, whereas serglycin carrying HS or HP is not capable for binding to CD44 (17, 55). Secreted serglycin in the tumor microenvironment may interact with CD44 on tumor cells triggering CD44 signaling (Figure 3). CD44 is involved in cell–cell and cell-matrix interactions and signals through several pathways by binding via its cytoplasmic domain to multiple cell membrane and intracellular functional proteins thus regulating cancer cells’ epithelial to mesenchymal transition (EMT), migration, metastasis, proliferation, apoptosis, and resistance (104, 105). Importantly, CD44 has been recognized as a cancer stem cell marker for a variety of tumor types.

The expression of serglycin was also confirmed in several MM cell lines (16). Serglycin is the major PG synthesized by MM cells and is constitutively secreted to the culture medium. Interestingly, serglycin is also localized on the cell surface where it is attached through its CS-4 chains. Serglycin levels are elevated in the bone marrow aspirates of patients with newly diagnosed MM, suggesting a potent correlation of serglycin accumulation with disease progression (16). Serglycin present on myeloma cell surface promotes the adhesion of myeloma cells to collagen I. The adhesion of myeloma cells to collagen I but also the interaction of soluble collagen I with myeloma cells via cell-surface serglycin enhances the biosynthesis and secretion of MMP2 and MMP9, which are involved in bone destruction (57) (Figure 3). The regulatory role of serglycin in the biosynthesis and secretion of proteases has been also shown in other cellular systems (23, 106). Serglycin is colocalized with tPA in secretory vesicles in HUVEC (23), whereas Madin-Darby canine kidney cells stably transfected with serglycin express elevated levels of MMP9 and urokinase plasminogen activator (uPA) both at mRNA and protein levels (106). The release of proteolytic enzymes by tumor cells or stromal cells and the regulation of their activity in the tumor microenvironment are crucial for tumor progression and tumor-induced bone disease (107, 108). In addition, secreted serglycin was found to influence the bone mineralization process through inhibition of the crystal growth rate of hydroxyapatite, thus providing another possible explanation for impaired bone formation and loss of bone mass commonly seen in MM patients (16). Serglycin synthesized and secreted by human acute monocytic leukemia cell line THP1 as well as serglycin isolated from myeloma cells forms complexes with proform of MMP9 (proMMP9) *in vivo* and *in vitro* (73, 74). Both the hemopexin-like (PEX) domain and the fibronectin-like (FnII) module of the enzyme are involved in the formation of the heteromer. The formation of heteromers alters the mode of activation of proMMP9 and the interaction of the enzyme with its substrates (73, 109). Another study supports the direct protein–protein interaction between serglycin and MMPs (19). It has been shown that serglycin is colocalized with MMP13 in cytoplasmic granules in chondrocytes interacting with a fragment of C-terminal domain of MMP13 that comprises the hinge and PEX domains (19). ProMMP9 in the heteromer is activated in the presence of Ca^2+^, although this cation stabilizes MMP9 without activating the single proenzyme. Ca^2+^ induces the cleavage of both the C-terminal PEX domain of the enzyme and the core protein of PG and the release of the activated enzyme. MM located within bone marrow and solid tumors, which metastasize in the bones, induce bone destruction and release Ca^2+^. The accumulation of serglycin within bone marrow in MM may be involved in the formation of heteromers with proMMP9 in the ECM triggering a Ca^2+^-induced activation of the enzyme (Figure 3).

Matrix secreted and cell-surface associated serglycin protects myeloma cells from complement system attack induced by immunotherapy, therefore promoting the survival of myeloma cells (56). Serglycin inhibits specifically the classical and the lectin pathways via binding to C1q and MBL and do not interfere with the alternative pathway (56) (Figure 3). The intact serglycin molecule is required for binding to MBL, whereas binding to C1q is mediated exclusively by CS-4 side chains. A similar mode of inhibition of the classical and lectin pathways was demonstrated for secreted serglycin by aggressive breast cancer cells (110). It has been shown previously that complement components such as C1q, C3, C3a, C4, C5, and the membrane attack complex (MAC) are deposited in the inflammatory tumor microenvironment. The assumption has been made is that these activated complement proteins play a role in tumor defense directly through complement-dependent cytotoxicity and indirectly through antibody-dependent cell-mediated cytotoxicity (111). The mechanism of complement activation in cancer is known to involve mainly the classical and the lectin pathways (111). Complement is activated by factors present on tumor cells or induced by treatment of tumor cells with therapeutic antibodies. Malignant cells express a variety of complement inhibitors, which all attenuate complement cytotoxicity (111). The inhibition of complement is also a great limitation during immunotherapy against several types of cancer. These data suggest a role for serglycin as a modulator of immune system response in the tumor microenvironment that may protect tumor cells from complement attack. Another great limitation in the treatment of malignancies is the development of drug resistance. Serglycin is among genes that over-expressed in six tumor cell lines of hematopoietic origin that resist in doxorubicin, methotrexate, cisplatin, and vincristine treatment compared to the drug sensitive parental cell lines (112). The implication of serglycin in drug resistance is of great interest and the mechanism of action is yet unknown.

Only few studies have demonstrated expression of serglycin in non-hematological tumors. Increased expression of serglycin in patients with hepatocellular and nasopharyngeal carcinoma was correlated with unfavorable prognosis and represented an independent unfavorable prognostic indicator for overall survival and recurrence as well as disease free and distant metastasis free survival (30, 103). Metastatic nasopharyngeal carcinoma cells highly express and secrete in the culture medium serglycin, which promotes motility, invasion, and metastasis (30). The overexpression of serglycin is also associated with EMT in nasopharyngeal cancer cells. The functions of serglycin were dependent on the fully glycosylated molecule. Treatment of cancer cells with exogenously added glycanated serglycin promoted cancer cell metastasis and invasion, whereas non-glycosylated core protein of serglycin failed to induce cell motility (30). *In vitro*, serglycin was highly expressed and secreted by aggressive tumor cell lines (110). In invasive MDA-MB-231 breast cancer cells serglycin represents the major PG type and is abundantly expressed and secreted in the culture medium (110). Furthermore, serglycin is highly expressed by other aggressive breast cancer cells, which also belong to the Basal B subgroup, and they show mesenchymal phenotype, enhanced invasive properties and enriched expression of EMT transcriptional drivers (113). These cells exhibit an EMT gene signature and are found to resemble breast cancer stem cells, being CD44^high^CD24^low^ (114). Interestingly, stable overexpression of intact serglycin gene and a truncated form of serglycin lacking GAG attachment sites in low aggressive MCF-7 breast cancer cells demonstrated that serglycin promotes breast cancer cell anchorage-independent growth, migration, and invasion. The tumor promoting properties of serglycin are dependent on the overexpression and secretion of glycanated serglycin (110). Therefore, the specific structure of CS-4 present on serglycin is important for serglycin functions in breast cancer. Altered biosynthesis of CS chains has been demonstrated in various cancer types. Specific structures of CS influence various biological processes during tumor growth and spread (115). CHST11 gene that specifically mediates 4-O sulfation of CS is highly expressed in MDA-MB-231 breast cancer cells and breast cancer tissues. CS-4 chains mediate the binding of breast cancer cells to P-selectin and facilitate the formation of metastasis (116).

## Inflammation Augments Malignancy: Possible Roles of Serglycin

During tumor development, cancer cells produce cytokines and chemokines that attract and activate inflammatory cells, endothelial cells, fibroblasts, and platelets to secrete growth factors, cytokines, and chemokines. Serglycin may regulate the biosynthesis, secretion, and targeted delivery of many inflammatory mediators, which can act in various cell types in paracrine and autocrine manner in multiple ways to enhance inflammatory process and support tumor growth and metastasis (Figure 3).

Tumors are often infiltrated by immune cells, such as T lymphocytes, mast cells, and macrophages, which are recruited to the site by chemokines and cytokines secreted by the various cells in the tumor milieu. Macrophages are attracted by responding to CCL2, IL-4, IL-10, and IL-13, acquire an activated phenotype with pro-tumorigenic properties, releasing a variety of chemokines, cytokines, growth factors, and proteases (117). Mast cells infiltration is occurred in the tumor microenvironment in a number of human malignancies in response to tumor-derived chemoattractants such as CCL2 and CCL5. The presence of mast cells was shown to correlate with either favorable or poor prognosis depending on the tumor. Mast cells can exert pro-tumorigenic effects by secreting factors like VEGF, angiopoietin-1, CXCL1, and IL-8 that promote tumor angiogenesis, as well as growth factors such as PDGF, NGF, SCF, FGF2, and proteases that facilitate tumor cell growth and metastases (118). Mast cell proteases exert dual roles in the regulation of inflammatory process. Several pro-inflammatory chemokines and cytokines are substrates of mast cell proteases and their cleavage results in the activation or inactivation of inflammatory mediators (119). Chymase also indirectly influences ECM remodeling via its ability to activate various MMPs (120, 121). Both tryptase and chymase are directly involved in ECM degradation. Tryptase degrades collagen type IV present in basement membranes while chymase cleaves vitronectin and procollagen and both degrade fibronectin (119).

Endothelial cells synthesize and secrete chemokines, cytokines, and growth factors such as CXCL1 and respond to inflammatory stimuli enhancing their production in a serglycin-depended manner (22). Cancer associated fibroblasts which are activated by cancer cells also overexpress inflammatory mediators as well as serglycin and a disintegrin and metalloproteinase with thrombospondin motifs 1 (ADAMTS1), promoting cancer cell invasion (24).

Considering that single GAG chains and serglycin interact with growth factors, cytokines, and chemokines (7, 122), it is plausible that serglycin secreted by cancer and stromal cells is important for the protection of inflammatory mediators in ECM and the creation of chemotactic gradients. Furthermore, serglycin may be involved in the presentation of these molecules to high affinity receptors thus enhancing signaling events (Figure 3).

Platelets have long been believed to play a critical role in cancer metastasis through the enhancement of circulating tumor cells survival and adhesion to the endothelium in the circulation (123). Serglycin may be involved in tumor cell metastasis either via its proven role in activation of platelets or directly affecting the binding of tumor cells to platelets (Figure 3). The presence of serglycin in platelets is critical for packaging and secretion of selected α-granule proteins. The reduced secretion of dense granule contents results in impaired platelet activation and aggregation (7, 96). Tumor cells may bind to the surface of activated platelets via platelet receptors glycoprotein IIb/IIIa (integrin αIIbβ3) and P-selectin or by attachment to platelets microparticles. This most likely requires the activation of platelets, the fusion of α-granule membrane with the cell membrane, the exposure of activation-induced surface proteins and the secretion of chemokines, cytokines, and growth factors (7). Platelet serglycin may influence the release of these factors in the tumor microenvironment, which promote tumor cell growth and metastasis. Platelet-derived TGF-β as well as direct platelet-tumor cell contacts synergistically activate the TGF-β/Smad and nuclear factor kappa-light-chain-enhancer of activated B cells (NF-κB) pathways in cancer cells, resulting in their transition to an invasive mesenchymal-like phenotype and enhanced metastasis *in vivo* (124) (Figure 3). Another possibility is that released serglycin either by tumor cells or platelets may participate in bridging tumor cells with activated platelets or platelet microparticles as well as endothelial cells. It is proposed that serglycin is bound on the surface of platelets (7), whereas serglycin has been identified on the surface of tumor cells (6, 16). Secreted serglycin substituted with CS chains may associate with tumor cell surface via CD44 (55) and the membrane of activated platelets and endothelial cells via P-selectin (116) and/or other unidentified receptors (Figure 3). The inhibition of platelets-tumor cell interaction has been targeted for treatment of metastasis. HP and other GAGs isolated from various sources prevent metastasis. Although the anti-metastatic effect of HP was initially believed to associate with its anticoagulant activity, later it was found that interfered with binding of activated platelets with ligands on tumor cells (125).

## Conclusion

Serglycin is a dominant PG in immune cells with a major impact on their biology. Numerous studies in the past using mainly a valuable serglycin knockdown mouse model demonstrated important functional roles for serglycin in immune system processes and inflammation. Recent studies have revealed emerging roles for serglycin in tumorigenesis. Collectively, the expression of serglycin seems to benefit tumor cells in multiple ways. It may act as a modulator of immune system in tumor microenvironment and enrich tumor cells with resistance to various therapeutic agents. Serglycin augments the invasion and metastasis of tumor cells with yet unknown molecular mechanisms. Importantly, it serves as an ideal molecular partner for multiple molecular effectors, such as proteolytic enzymes, chemokines, cytokines, and growth factors regulating their biosynthesis and secretion or/and enhancing their activities by protecting and accompanying them to specific target sites.

## Conflict of Interest Statement

The authors declare that the research was conducted in the absence of any commercial or financial relationships that could be construed as a potential conflict of interest.

## References

[1] Theocharis AD Skandalis SS Tzanakakis GN Karamanos NK Proteoglycans in health and disease: novel roles for proteoglycans in malignancy and their pharmacological targeting. FEBS J (2010) 277(19):3904–23 10.1111/j.1742-4658.2010.07800.x 20840587

[2] Iozzo RV Sanderson RD Proteoglycans in cancer biology, tumour microenvironment and angiogenesis. J Cell Mol Med (2011) 15(5):1013–31 10.1111/j.1582-4934.2010.01236.x 21155971 PMC3633488

[3] Afratis N Gialeli C Nikitovic D Tsegenidis T Karousou E Theocharis AD Glycosaminoglycans: key players in cancer cell biology and treatment. FEBS J (2012) 279(7):1177–97 10.1111/j.1742-4658.2012.08529.x 22333131

[4] Kolset SO Pejler G Serglycin: a structural and functional chameleon with wide impact on immune cells. J Immunol (2011) 187(10):4927–33 10.4049/jimmunol.1100806 22049227

[5] Scully OJ Chua PJ Harve KS Bay BH Yip GW Serglycin in health and diseases. Anat Rec (Hoboken) (2012) 295(9):1415–20 10.1002/ar.22536 22807344

[6] Bourdon MA Oldberg A Pierschbacher M Ruoslahti E Molecular cloning and sequence analysis of a chondroitin sulfate proteoglycan cDNA. Proc Natl Acad Sci U S A (1985) 82(5):1321–5 10.1073/pnas.82.5.1321 3919394 PMC397252

[7] Schick BP Serglycin proteoglycan deletion in mouse platelets: physiological effects and their implications for platelet contributions to thrombosis, inflammation, atherosclerosis, and metastasis. Prog Mol Biol Transl Sci (2010) 93:235–87 10.1016/S1877-1173(10)93011-1 20807648

[8] Kolset SO Gallagher JT Proteoglycans in haemopoietic cells. Biochim Biophys Acta (1990) 1032(2–3):191–211 2261494 10.1016/0304-419x(90)90004-k

[9] Maillet P Alliel PM Mitjavila MT Perin JP Jolles P Bonnet F Expression of the serglycin gene in human leukemic cell lines. Leukemia (1992) 6(11):1143–7 1434796

[10] Niemann CU Kjeldsen L Ralfkiaer E Jensen MK Borregaard N Serglycin proteoglycan in hematologic malignancies: a marker of acute myeloid leukemia. Leukemia (2007) 21(12):2406–10 10.1038/sj.leu.2404975 17928883

[11] Oynebraten I Hansen B Smedsrod B Uhlin-Hansen L Serglycin secreted by leukocytes is efficiently eliminated from the circulation by sinusoidal scavenger endothelial cells in the liver. J Leukoc Biol (2000) 67(2):183–8 10670578 10.1002/jlb.67.2.183

[12] Schick BP Jacoby JA Serglycin and betaglycan proteoglycans are expressed in the megakaryocytic cell line CHRF 288-11 and normal human megakaryocytes. J Cell Physiol (1995) 165(1):96–106 10.1002/jcp.1041650113 7559813

[13] Schick BP Senkowski-Richardson S Proteoglycan synthesis in human erythroleukaemia (HEL) cells. Biochem J (1992) 282(Pt 3):651–8 1372801 10.1042/bj2820651 PMC1130837

[14] Stellrecht CM Fraizer G Selvanayagam C Chao LY Lee A Saunders GF Transcriptional regulation of a hematopoietic proteoglycan core protein gene during hematopoiesis. J Biol Chem (1993) 268(6):4078–84 8440699

[15] Stellrecht CM Mars WM Miwa H Beran M Saunders GF Expression pattern of a hematopoietic proteoglycan core protein gene during human hematopoiesis. Differentiation (1991) 48(2):127–35 10.1111/j.1432-0436.1991.tb00251.x 1723052

[16] Theocharis AD Seidel C Borset M Dobra K Baykov V Labropoulou V Serglycin constitutively secreted by myeloma plasma cells is a potent inhibitor of bone mineralization in vitro. J Biol Chem (2006) 281(46):35116–28 10.1074/jbc.M601061200 16870619

[17] Toyama-Sorimachi N Kitamura F Habuchi H Tobita Y Kimata K Miyasaka M Widespread expression of chondroitin sulfate-type serglycins with CD44 binding ability in hematopoietic cells. J Biol Chem (1997) 272(42):26714–9 10.1074/jbc.272.42.26714 9334256

[18] Biederbick A Licht A Kleene R Serglycin proteoglycan is sorted into zymogen granules of rat pancreatic acinar cells. Eur J Cell Biol (2003) 82(1):19–29 10.1078/0171-9335-00287 12602945

[19] Zhang L Yang M Yang D Cavey G Davidson P Gibson G Molecular interactions of MMP-13 C-terminal domain with chondrocyte proteins. Connect Tissue Res (2010) 51(3):230–9 10.3109/03008200903288902 20073988

[20] Kulseth MA Kolset SO Ranheim T Stimulation of serglycin and CD44 mRNA expression in endothelial cells exposed to TNF-alpha and IL-1alpha. Biochim Biophys Acta (1999) 1428(2–3):225–32 10.1016/S0304-4165(99)00096-3 10434040

[21] Lemire JM Chan CK Bressler S Miller J LeBaron RG Wight TN Interleukin-1beta selectively decreases the synthesis of versican by arterial smooth muscle cells. J Cell Biochem (2007) 101(3):753–66 10.1002/jcb.21235 17226775

[22] Meen AJ Oynebraten I Reine TM Duelli A Svennevig K Pejler G Serglycin is a major proteoglycan in polarized human endothelial cells and is implicated in the secretion of the chemokine GROalpha/CXCL1. J Biol Chem (2011) 286(4):2636–47 10.1074/jbc.M110.151944 21075844 PMC3024759

[23] Schick BP Gradowski JF San Antonio JD Synthesis, secretion, and subcellular localization of serglycin proteoglycan in human endothelial cells. Blood (2001) 97(2):449–58 10.1182/blood.V97.2.449 11154222

[24] Tyan SW Hsu CH Peng KL Chen CC Kuo WH Lee EY Breast cancer cells induce stromal fibroblasts to secrete ADAMTS1 for cancer invasion through an epigenetic change. PLoS One (2012) 7(4):e35128 10.1371/journal.pone.0035128 22514714 PMC3325931

[25] Werth BB Bashir M Chang L Werth VP Ultraviolet irradiation induces the accumulation of chondroitin sulfate, but not other glycosaminoglycans, in human skin. PLoS One (2011) 6(8):e14830 10.1371/journal.pone.0014830 21829593 PMC3150335

[26] Grover A Edwards SA Bourdon M Adamson ED Proteoglycan-19, laminin and collagen type IV production is correlated with the levels of mRNA in F9 cell aggregates differentiating in the presence or absence of cyclic AMP. Differentiation (1987) 36(2):138–44 10.1111/j.1432-0436.1987.tb00188.x 2834254

[27] Gasimli L Stansfield HE Nairn AV Liu H Paluh JL Yang B Structural remodeling of proteoglycans upon retinoic acid-induced differentiation of NCCIT cells. Glycoconj J (2013) 30(5):497–510 10.1007/s10719-012-9450-x 23053635 PMC3556377

[28] Schick BP Ho HC Brodbeck KC Wrigley CW Klimas J Serglycin proteoglycan expression and synthesis in embryonic stem cells. Biochim Biophys Acta (2003) 1593(2–3):259–67 10.1016/S0167-4889(02)00396-8 12581870

[29] Keith Ho HC McGrath KE Brodbeck KC Palis J Schick BP Serglycin proteoglycan synthesis in the murine uterine decidua and early embryo. Biol Reprod (2001) 64(6):1667–76 10.1095/biolreprod64.6.1667 11369593

[30] Li XJ Ong CK Cao Y Xiang YQ Shao JY Ooi A Serglycin is a theranostic target in nasopharyngeal carcinoma that promotes metastasis. Cancer Res (2011) 71(8):3162–72 10.1158/0008-5472.CAN-10-3557 21289131

[31] Stevens RL Fox CC Lichtenstein LM Austen KF Identification of chondroitin sulfate E proteoglycans and heparin proteoglycans in the secretory granules of human lung mast cells. Proc Natl Acad Sci U S A (1988) 85(7):2284–7 10.1073/pnas.85.7.2284 3353378 PMC279975

[32] Kjellen L Pettersson I Lillhager P Steen ML Pettersson U Lehtonen P Primary structure of a mouse mastocytoma proteoglycan core protein. Biochem J (1989) 263(1):105–13 2532501 10.1042/bj2630105 PMC1133396

[33] Lidholt K Eriksson I Kjellen L Heparin proteoglycans synthesized by mouse mastocytoma contain chondroitin sulphate. Biochem J (1995) 311(Pt 1):233–8 7575459 10.1042/bj3110233 PMC1136143

[34] Abrink M Grujic M Pejler G Serglycin is essential for maturation of mast cell secretory granule. J Biol Chem (2004) 279(39):40897–905 10.1074/jbc.M405856200 15231821

[35] Yurt RW Leid RW Jr Austen KF Native heparin from rat peritoneal mast cells. J Biol Chem (1977) 252(2):518–21 833141

[36] Gilead L Livni N Eliakim R Ligumsky M Fich A Okon E Human gastric mucosal mast cells are chondroitin sulphate E-containing mast cells. Immunology (1987) 62(1):23–8 3115893 PMC1453708

[37] Enerback L Kolset SO Kusche M Hjerpe A Lindahl U Glycosaminoglycans in rat mucosal mast cells. Biochem J (1985) 227(2):661–8 4004785 10.1042/bj2270661 PMC1144886

[38] Stevens RL Lee TD Seldin DC Austen KF Befus AD Bienenstock J Intestinal mucosal mast cells from rats infected with *Nippostrongylus brasiliensis* contain protease-resistant chondroitin sulfate di-B proteoglycans. J Immunol (1986) 137(1):291–5 3086452

[39] Murata K Acidic glycosaminoglycans in human platelets and leukocytes: the isolation and enzymatic characterization of chondroitin 4-sulfate. Clin Chim Acta (1974) 57(2):115–24 10.1016/0009-8981(74)90418-5 4279788

[40] Olsson I Gardell S Isolation and characterization of glycosaminoglycans from human leukocytes and platelets. Biochim Biophys Acta (1967) 141(2):348–57 10.1016/0304-4165(67)90109-2 6057662

[41] Metcalfe DD Wasserman SI Austen KF Isolation and characterization of sulphated mucopolysaccharides from rat leukaemic (RBL-1) basophils. Biochem J (1980) 185(2):367–72 6446899 10.1042/bj1850367 PMC1161362

[42] Orenstein NS Galli SJ Dvorak AM Silbert JE Dvorak HF Sulfated glycosaminoglycans of guinea pig basophilic leukocytes. J Immunol (1978) 121(2):586–92 681751

[43] Schick BP Walsh CJ Jenkins-West T Sulfated proteoglycans and sulfated proteins in guinea pig megakaryocytes and platelets in vivo. Relevance to megakaryocyte maturation and platelet activation. J Biol Chem (1988) 263(2):1052–62 3335514

[44] Kolset SO Oversulfated chondroitin sulfate proteoglycan in cultured human peritoneal macrophages. Biochem Biophys Res Commun (1986) 139(2):377–82 10.1016/S0006-291X(86)80001-8 3767967

[45] Kolset SO Kjellen L Seljelid R Lindahl U Changes in glycosaminoglycan biosynthesis during differentiation in vitro of human monocytes. Biochem J (1983) 210(3):661–7 6870801 10.1042/bj2100661 PMC1154275

[46] Uhlin-Hansen L Eskeland T Kolset SO Modulation of the expression of chondroitin sulfate proteoglycan in stimulated human monocytes. J Biol Chem (1989) 264(25):14916–22 2768247

[47] Mattei MG Perin JP Alliel PM Bonnet F Maillet P Passage E Localization of human platelet proteoglycan gene to chromosome 10, band q22.1, by in situ hybridization. Hum Genet (1989) 82(1):87–8 10.1007/BF00288281 2714783

[48] Stevens RL Avraham S Gartner MC Bruns GA Austen KF Weis JH Isolation and characterization of a cDNA that encodes the peptide core of the secretory granule proteoglycan of human promyelocytic leukemia HL-60 cells. J Biol Chem (1988) 263(15):7287–91 2835370

[49] Humphries DE Nicodemus CF Schiller V Stevens RL The human serglycin gene. Nucleotide sequence and methylation pattern in human promyelocytic leukemia HL-60 cells and T-lymphoblast Molt-4 cells. J Biol Chem (1992) 267(19):13558–63 1377686

[50] Nicodemus CF Avraham S Austen KF Purdy S Jablonski J Stevens RL Characterization of the human gene that encodes the peptide core of secretory granule proteoglycans in promyelocytic leukemia HL-60 cells and analysis of the translated product. J Biol Chem (1990) 265(10):5889–96 2180935

[51] Schick BP Petrushina I Brodbeck KC Castronuevo P Promoter regulatory elements and DNase I-hypersensitive sites involved in serglycin proteoglycan gene expression in human erythroleukemia, CHRF 288-11, and HL-60 cells. J Biol Chem (2001) 276(27):24726–35 10.1074/jbc.M102958200 11333275

[52] Papas TS Watson DK Sacchi N Fujiwara S Seth AK Fisher RJ ETS family of genes in leukemia and Down syndrome. Am J Med Genet Suppl (1990) 7:251–61 2149958 10.1002/ajmg.1320370751

[53] Deininger P Alu elements: know the SINEs. Genome Biol (2011) 12(12):236 10.1186/gb-2011-12-12-236 22204421 PMC3334610

[54] Castronuevo P Thornton MA McCarthy LE Klimas J Schick BP DNase I hypersensitivity patterns of the serglycin proteoglycan gene in resting and phorbol 12-myristate 13-acetate-stimulated human erythroleukemia (HEL), CHRF 288-11, and HL-60 cells compared with neutrophils and human umbilical vein endothelial cells. J Biol Chem (2003) 278(49):48704–12 10.1074/jbc.M310220200 14506241

[55] Toyama-Sorimachi N Sorimachi H Tobita Y Kitamura F Yagita H Suzuki K A novel ligand for CD44 is serglycin, a hematopoietic cell lineage-specific proteoglycan. Possible involvement in lymphoid cell adherence and activation. J Biol Chem (1995) 270(13):7437–44 10.1074/jbc.270.13.7437 7535771

[56] Skliris A Happonen KE Terpos E Labropoulou V Borset M Heinegard D Serglycin inhibits the classical and lectin pathways of complement via its glycosaminoglycan chains: implications for multiple myeloma. Eur J Immunol (2011) 41(2):437–49 10.1002/eji.201040429 21268013

[57] Skliris A Labropoulou VT Papachristou DJ Aletras A Karamanos NK Theocharis AD Cell-surface serglycin promotes adhesion of myeloma cells to collagen type I and affects the expression of matrix metalloproteinases. FEBS J (2013) 280(10):2342–52 10.1111/febs.12179 23387827

[58] Brennan MJ Oldberg A Hayman EG Ruoslahti E Effect of a proteoglycan produced by rat tumor cells on their adhesion to fibronectin-collagen substrata. Cancer Res (1983) 43(9):4302–7 6683588

[59] Schick BP Pestina TI San Antonio JD Stenberg PE Jackson CW Decreased serglycin proteoglycan size is associated with the plateletalpha granule storage defect in Wistar Furth hereditary macrothrombocytopenic rats. Serglycin binding affinity to type I collagen is unaltered. J Cell Physiol (1997) 172(1):87–93 10.1002/(SICI)1097-4652(199707)172:1<87::AID-JCP10>3.0.CO;2-L 9207929

[60] Kolset SO Mann DM Uhlin-Hansen L Winberg JO Ruoslahti E Serglycin-binding proteins in activated macrophages and platelets. J Leukoc Biol (1996) 59(4):545–54 8613703 10.1002/jlb.59.4.545

[61] Galvin JP Spaeny-Dekking LH Wang B Seth P Hack CE Froelich CJ Apoptosis induced by granzyme B-glycosaminoglycan complexes: implications for granule-mediated apoptosis in vivo. J Immunol (1999) 162(9): 5345–50 10228010

[62] Metkar SS Wang B Aguilar-Santelises M Raja SM Uhlin-Hansen L Podack E Cytotoxic cell granule-mediated apoptosis: perforin delivers granzyme B-serglycin complexes into target cells without plasma membrane pore formation. Immunity (2002) 16(3):417–28 10.1016/S1074-7613(02)00286-8 11911826

[63] Raja SM Wang B Dantuluri M Desai UR Demeler B Spiegel K Cytotoxic cell granule-mediated apoptosis. Characterization of the macromolecular complex of granzyme B with serglycin. J Biol Chem (2002) 277(51):49523–30 10.1074/jbc.M209607200 12388539

[64] Pejler G Maccarana M Interaction of heparin with rat mast cell protease 1. J Biol Chem (1994) 269(20):14451–6 8182050

[65] Kolset SO Prydz K Pejler G Intracellular proteoglycans. Biochem J (2004) 379(Pt 2):217–27 10.1042/BJ20031230 14759226 PMC1224092

[66] Lindstedt KA Kokkonen JO Kovanen PT Regulation of the activity of secreted human lung mast cell tryptase by mast cell proteoglycans. Biochim Biophys Acta (1998) 1425(3):617–27 10.1016/S0304-4165(98)00115-9 9838225

[67] Stevens RL Adachi R Protease-proteoglycan complexes of mouse and human mast cells and importance of their beta-tryptase-heparin complexes in inflammation and innate immunity. Immunol Rev (2007) 217:155–67 10.1111/j.1600-065X.2007.00525.x 17498058

[68] Braga T Grujic M Lukinius A Hellman L Abrink M Pejler G Serglycin proteoglycan is required for secretory granule integrity in mucosal mast cells. Biochem J (2007) 403(1):49–57 10.1042/BJ20061257 17147513 PMC1828881

[69] Ringvall M Ronnberg E Wernersson S Duelli A Henningsson F Abrink M Serotonin and histamine storage in mast cell secretory granules is dependent on serglycin proteoglycan. J Allergy Clin Immunol (2008) 121(4):1020–6 10.1016/j.jaci.2007.11.031 18234316

[70] Ronnberg E Calounova G Pejler G Mast cells express tyrosine hydroxylase and store dopamine in a serglycin-dependent manner. Biol Chem (2012) 393(1–2):107–12 10.1515/BC-2011-220 22628305

[71] Niemann CU Abrink M Pejler G Fischer RL Christensen EI Knight SD Neutrophil elastase depends on serglycin proteoglycan for localization in granules. Blood (2007) 109(10):4478–86 10.1182/blood-2006-02-001719 17272511

[72] Lemansky P Smolenova E Wrocklage C Hasilik A Neutrophil elastase is associated with serglycin on its way to lysosomes in U937 cells. Cell Immunol (2007) 246(1):1–7 10.1016/j.cellimm.2007.06.001 17617393

[73] Malla N Berg E Theocharis AD Svineng G Uhlin-Hansen L Winberg JO In vitro reconstitution of complexes between pro-matrix metalloproteinase-9 and the proteoglycans serglycin and versican. FEBS J (2013) 280(12):2870–87 10.1111/febs.12291 23601700

[74] Winberg JO Kolset SO Berg E Uhlin-Hansen L Macrophages secrete matrix metalloproteinase 9 covalently linked to the core protein of chondroitin sulphate proteoglycans. J Mol Biol (2000) 304(4):669–80 10.1006/jmbi.2000.4235 11099388

[75] Vinayagam A Stelzl U Foulle R Plassmann S Zenkner M Timm J A directed protein interaction network for investigating intracellular signal transduction. Sci Signal (2011) 4(189):rs8 10.1126/scisignal.2001699 21900206

[76] Lim J Hao T Shaw C Patel AJ Szabo G Rual JF A protein-protein interaction network for human inherited ataxias and disorders of Purkinje cell degeneration. Cell (2006) 125(4):801–14 10.1016/j.cell.2006.03.032 16713569

[77] Rual JF Venkatesan K Hao T Hirozane-Kishikawa T Dricot A Li N Towards a proteome-scale map of the human protein-protein interaction network. Nature (2005) 437(7062):1173–8 10.1038/nature04209 16189514

[78] Jang CY Wong J Coppinger JA Seki A Yates JR III Fang G DDA3 recruits microtubule depolymerase Kif2a to spindle poles and controls spindle dynamics and mitotic chromosome movement. J Cell Biol (2008) 181(2):255–67 10.1083/jcb.200711032 18411309 PMC2315673

[79] Kumar A Rajendran V Sethumadhavan R Purohit R CEP proteins: the knights of centrosome dynasty. Protoplasma (2013) 250(5):965–83 10.1007/s00709-013-0488-9 23456457

[80] Leznicki P High S SGTA antagonizes BAG6-mediated protein triage. Proc Natl Acad Sci U S A (2012) 109(47):19214–9 10.1073/pnas.1209997109 23129660 PMC3511132

[81] Akahane T Sahara K Yashiroda H Tanaka K Murata S Involvement of Bag6 and the TRC pathway in proteasome assembly. Nat Commun (2013) 4:2234 10.1038/ncomms3234 23900548

[82] Kim ST Tasaki T Zakrzewska A Yoo YD Sa Sung K Kim BY The N-end rule proteolytic system in autophagy. Autophagy (2013) 9(7):10.4161/auto.24643 23628846 PMC3722320

[83] Tasaki T Kim ST Zakrzewska A Lee BE Kang MJ Yoo YD UBR box N-recognin-4 (UBR4), an N-recognin of the N-end rule pathway, and its role in yolk sac vascular development and autophagy. Proc Natl Acad Sci U S A (2013) 110(10):3800–5 10.1073/pnas.1217358110 23431188 PMC3593856

[84] Su V Nakagawa R Koval M Lau AF Ubiquitin-independent proteasomal degradation of endoplasmic reticulum-localized connexin43 mediated by CIP75. J Biol Chem (2010) 285(52):40979–90 10.1074/jbc.M110.170753 20940304 PMC3003397

[85] Yun Lee D Arnott D Brown EJ Ubiquilin4 is an adaptor protein that recruits Ubiquilin1 to the autophagy machinery. EMBO Rep (2013) 14(4):373–81 10.1038/embor.2013.22 23459205 PMC3615663

[86] Wu G Feng X Stein L A human functional protein interaction network and its application to cancer data analysis. Genome Biol (2010) 11(5):R53 10.1186/gb-2010-11-5-r53 20482850 PMC2898064

[87] Zernichow L Abrink M Hallgren J Grujic M Pejler G Kolset SO Serglycin is the major secreted proteoglycan in macrophages and has a role in the regulation of macrophage tumor necrosis factor-alpha secretion in response to lipopolysaccharide. J Biol Chem (2006) 281(37):26792–801 10.1074/jbc.M512889200 16807245

[88] Imoto-Tsubakimoto H Takahashi T Ueyama T Ogata T Adachi A Nakanishi N Serglycin is a novel adipocytokine highly expressed in epicardial adipose tissue. Biochem Biophys Res Commun (2013) 432(1):105–10 10.1016/j.bbrc.2013.01.078 23376071

[89] Pejler G Sadler JE Mechanism by which heparin proteoglycan modulates mast cell chymase activity. Biochemistry (1999) 38(37):12187–95 10.1021/bi991046b 10508424

[90] Pejler G Berg L Regulation of rat mast cell protease 1 activity. Protease inhibition is prevented by heparin proteoglycan. Eur J Biochem (1995) 233(1):192–9 10.1111/j.1432-1033.1995.192_1.x 7588746

[91] Lindstedt L Lee M Kovanen PT Chymase bound to heparin is resistant to its natural inhibitors and capable of proteolyzing high density lipoproteins in aortic intimal fluid. Atherosclerosis (2001) 155(1):87–97 10.1016/S0021-9150(00)00544-X 11223430

[92] Hallgren J Spillmann D Pejler G Structural requirements and mechanism for heparin-induced activation of a recombinant mouse mast cell tryptase, mouse mast cell protease-6: formation of active tryptase monomers in the presence of low molecular weight heparin. J Biol Chem (2001) 276(46):42774–81 10.1074/jbc.M105531200 11533057

[93] Sakai K Ren S Schwartz LB A novel heparin-dependent processing pathway for human tryptase. Autocatalysis followed by activation with dipeptidyl peptidase I. J Clin Invest (1996) 97(4):988–95 10.1172/JCI118523 8613553 PMC507145

[94] Kolset SO Tveit H Serglycin – structure and biology. Cell Mol Life Sci (2008) 65(7–8):1073–85 10.1007/s00018-007-7455-6 18066495 PMC11131666

[95] Grujic M Braga T Lukinius A Eloranta ML Knight SD Pejler G Serglycin-deficient cytotoxic T lymphocytes display defective secretory granule maturation and granzyme B storage. J Biol Chem (2005) 280(39):33411–8 10.1074/jbc.M501708200 16046402

[96] Woulfe DS Lilliendahl JK August S Rauova L Kowalska MA Abrink M Serglycin proteoglycan deletion induces defects in platelet aggregation and thrombus formation in mice. Blood (2008) 111(7):3458–67 10.1182/blood-2007-07-104703 18094327 PMC2275015

[97] Melo FR Waern I Ronnberg E Abrink M Lee DM Schlenner SM A role for serglycin proteoglycan in mast cell apoptosis induced by a secretory granule-mediated pathway. J Biol Chem (2011) 286(7):5423–33 10.1074/jbc.M110.176461 21123167 PMC3037655

[98] Melo FR Grujic M Spirkoski J Calounova G Pejler G Serglycin proteoglycan promotes apoptotic versus necrotic cell death in mast cells. J Biol Chem (2012) 287(22):18142–52 10.1074/jbc.M112.344796 22493512 PMC3365731

[99] Grujic M Christensen JP Sorensen MR Abrink M Pejler G Thomsen AR Delayed contraction of the CD8+ T cell response toward lymphocytic choriomeningitis virus infection in mice lacking serglycin. J Immunol (2008) 181(2):1043–51 18606656 10.4049/jimmunol.181.2.1043

[100] Sawesi O Spillmann D Lunden A Wernersson S Abrink M Serglycin-independent release of active mast cell proteases in response to *Toxoplasma gondii* infection. J Biol Chem (2010) 285(49):38005–13 10.1074/jbc.M110.118471 20864536 PMC2992234

[101] Kim JS Werth VP Identification of specific chondroitin sulfate species in cutaneous autoimmune disease. J Histochem Cytochem (2011) 59(8):780–90 10.1369/0022155411411304 21804080 PMC3261606

[102] Korpetinou A Milia-Argeiti E Labropoulou V Theocharis AD Serglycin: a novel player in the terrain of neoplasia. In: Karamanos NK, editor. Extracellular Matrix: Pathobiology and Signaling. Berlin: Walter de Gruyter GmbH & Co. KG (2012). p. 677–88

[103] He L Zhou X Qu C Tang Y Zhang Q Hong J Serglycin (SRGN) overexpression predicts poor prognosis in hepatocellular carcinoma patients. Med Oncol (2013) 30(4):707 10.1007/s12032-013-0707-4 23996242

[104] Zoller M CD44: can a cancer-initiating cell profit from an abundantly expressed molecule? Nat Rev Cancer (2011) 11(4):254–67 10.1038/nrc3023 21390059

[105] Skandalis SS Kozlova I Engstrom U Hellman U Heldin P Proteomic identification of CD44 interacting proteins. IUBMB Life (2010) 62(11):833–40 10.1002/iub.392 21117172

[106] Zernichow L Dalen KT Prydz K Winberg JO Kolset SO Secretion of proteases in serglycin transfected Madin-Darby canine kidney cells. FEBS J (2006) 273(3):536–47 10.1111/j.1742-4658.2005.05085.x 16420477

[107] Gialeli C Theocharis AD Karamanos NK Roles of matrix metalloproteinases in cancer progression and their pharmacological targeting. FEBS J (2011) 278(1):16–27 10.1111/j.1742-4658.2010.07919.x 21087457

[108] Labropoulou VT Theocharis AD Symeonidis A Skandalis SS Karamanos NK Kalofonos HP Pathophysiology and pharmacological targeting of tumor-induced bone disease: current status and emerging therapeutic interventions. Curr Med Chem (2011) 18(11):1584–98 10.2174/092986711795471275 21428887

[109] Winberg JO Berg E Kolset SO Uhlin-Hansen L Calcium-induced activation and truncation of promatrix metalloproteinase-9 linked to the core protein of chondroitin sulfate proteoglycans. Eur J Biochem (2003) 270(19):3996–4007 10.1046/j.1432-1033.2003.03788.x 14511382

[110] Korpetinou A Skandalis SS Moustakas A Happonen KE Tveit H Prydz K Serglycin is implicated in the promotion of aggressive phenotype of breast cancer cells. PLoS One (2013) 8(10):e78157 10.1371/journal.pone.0078157 24205138 PMC3815026

[111] Rutkowski MJ Sughrue ME Kane AJ Mills SA Parsa AT Cancer and the complement cascade. Mol Cancer Res (2010) 8(11):1453–65 10.1158/1541-7786.MCR-10-0225 20870736

[112] Beyer-Sehlmeyer G Hiddemann W Wormann B Bertram J Suppressive subtractive hybridisation reveals differential expression of serglycin, sorcin, bone marrow proteoglycan and prostate-tumour-inducing gene I (PTI-1) in drug-resistant and sensitive tumour cell lines of haematopoetic origin. Eur J Cancer (1999) 35(12):1735–42 10.1016/S0959-8049(99)00202-6 10674022

[113] Neve RM Chin K Fridlyand J Yeh J Baehner FL Fevr T A collection of breast cancer cell lines for the study of functionally distinct cancer subtypes. Cancer Cell (2006) 10(6):515–27 10.1016/j.ccr.2006.10.008 17157791 PMC2730521

[114] Blick T Hugo H Widodo E Waltham M Pinto C Mani SA Epithelial mesenchymal transition traits in human breast cancer cell lines parallel the CD44(hi/)CD24 (lo/-) stem cell phenotype in human breast cancer. J Mammary Gland Biol Neoplasia (2010) 15(2):235–52 10.1007/s10911-010-9175-z 20521089

[115] Theocharis AD Tsolakis I Tzanakakis GN Karamanos NK Chondroitin sulfate as a key molecule in the development of atherosclerosis and cancer progression. Adv Pharmacol (2006) 53:281–95 10.1016/S1054-3589(05)53013-8 17239771

[116] Cooney CA Jousheghany F Yao-Borengasser A Phanavanh B Gomes T Kieber-Emmons AM Chondroitin sulfates play a major role in breast cancer metastasis: a role for CSPG4 and CHST11 gene expression in forming surface P-selectin ligands in aggressive breast cancer cells. Breast Cancer Res (2011) 13(3):R58 10.1186/bcr2895 21658254 PMC3218947

[117] Evans A Costello E The role of inflammatory cells in fostering pancreatic cancer cell growth and invasion. Front Physiol (2012) 3:270 10.3389/fphys.2012.00270 22969725 PMC3431795

[118] Protti MP De Monte L Immune infiltrates as predictive markers of survival in pancreatic cancer patients. Front Physiol (2013) 4:210 10.3389/fphys.2013.00210 23950747 PMC3738865

[119] Dai H Korthuis RJ Mast cell proteases and inflammation. Drug Discov Today Dis Models (2011) 8(1):47–55 10.1016/j.ddmod.2011.06.004 22125569 PMC3223931

[120] Tchougounova E Lundequist A Fajardo I Winberg JO Abrink M Pejler G A key role for mast cell chymase in the activation of pro-matrix metalloprotease-9 and pro-matrix metalloprotease-2. J Biol Chem (2005) 280(10):9291–6 10.1074/jbc.M410396200 15615702

[121] Saarinen J Kalkkinen N Welgus HG Kovanen PT Activation of human interstitial procollagenase through direct cleavage of the Leu83-Thr84 bond by mast cell chymase. J Biol Chem (1994) 269(27):18134–40 8027075

[122] Zhang L Glycosaminoglycan (GAG) biosynthesis and GAG-binding proteins. Prog Mol Biol Transl Sci (2010) 93:1–17 10.1016/S1877-1173(10)93001-9 20807638

[123] Gay LJ Felding-Habermann B Platelets alter tumor cell attributes to propel metastasis: programming in transit. Cancer Cell (2011) 20(5):553–4 10.1016/j.ccr.2011.11.001 22094248

[124] Labelle M Begum S Hynes RO Direct signaling between platelets and cancer cells induces an epithelial-mesenchymal-like transition and promotes metastasis. Cancer Cell (2011) 20(5):576–90 10.1016/j.ccr.2011.09.009 22094253 PMC3487108

[125] Li J King MR Adhesion receptors as therapeutic targets for circulating tumor cells. Front Oncol (2012) 2:79 10.3389/fonc.2012.00079 22837985 PMC3402858

